# Therapeutic strategies targeting folate receptor α for ovarian cancer

**DOI:** 10.3389/fimmu.2023.1254532

**Published:** 2023-08-30

**Authors:** Jia Mai, Limei Wu, Ling Yang, Ting Sun, Xiaojuan Liu, Rutie Yin, Yongmei Jiang, Jinke Li, Qintong Li

**Affiliations:** ^1^ Department of Laboratory Medicine, Obstetrics & Gynecology and Pediatrics, West China Second University Hospital, Key Laboratory of Birth Defects and Related Diseases of Women and Children, Ministry of Education, Development and Related Diseases of Women and Children Key Laboratory of Sichuan Province, Center of Growth, Metabolism and Aging, State Key Laboratory of Biotherapy and Collaborative Innovation Center of Biotherapy, Sichuan University, Chengdu, Sichuan, China; ^2^ Department of Obstetrics and Gynecology, Chengdu Second People's Hospital, Chengdu, Sichuan, China; ^3^ Department of Clinical Laboratory, The first Affiliated Hospital of Zhengzhou University, Zhengzhou, China

**Keywords:** ovarian cancer, folate receptor α, FOLR1, mirvetuximab soravtansine, MIRV, Elahere, antibody-drug conjugate, ADC

## Abstract

Epithelial ovarian cancer (EOC) is the deadliest gynecological cancer, and presents a major clinical challenge due to limited treatment options. Folate receptor alpha (FRα), encoded by the FOLR1 gene, is an attractive therapeutically target due to its prevalent and high expression in EOC cells. Recent basic and translational studies have explored several modalities, such as antibody-drug conjugate (ADC), monoclonal antibodies, small molecules, and folate-drug conjugate, to exploit FRα for EOC treatment. In this review, we summarize the function of FRα, and clinical efficacies of various FRα-based therapeutics. We highlight mirvetuximab soravtansine (MIRV), or Elahere (ImmunoGen), the first FRα-targeting ADC approved by the FDA to treat platinum-resistant ovarian cancer. We discuss potential mechanisms and management of ocular adverse events associated with MIRV administration.

## 1 Introduction

Epithelial ovarian cancer (EOC) accounts for approximately 95% of ovarian cancer incidence, and is a leading cause of gynecologic cancer mortality worldwide (1, 2). Current standard-of-care treatment for newly diagnosed patients is cytoreductive debulking surgery plus neoadjuvant or post-operative platinum-based chemotherapy. Most patients initially respond to chemotherapy, but unfortunately up to 80% will eventually relapse leading to patient demise (3). Thus, platinum resistance presents a major clinical challenge. Angiogenesis inhibitor (bevacizumab) and the poly (ADP-ribose) polymerase inhibitors (olaparib, rucaparib and niraparib) provide some benefits for a subset of patients, but can only delay the relapse of platinum-resistant EOC (4, 5). Notably, recent large-scale clinical trials using immune-checkpoint inhibitors (anti-PD1/L1 monoclonal antibodies) failed to provide clinical benefit in EOC. In the past decades, the 5-year relative survival rates of ovarian cancer have only been moderately improved, from 43% in 1995 to 50% in 2018 in the USA (6, 7). Thus, treatment options for platinum-resistant EOC patients are limited, and present a major unmet clinical need.

Folate receptor alpha (FRα), encoded by the FOLR1 gene, has attracted considerable interest due to its high expression in several cancer types including those of lung and breast. FRα shows restricted tissue expression on the plasma membrane of epithelial cells in kidney, lung, ovary, fallopian tube, uterus, cervix, epididymis and placenta, and is highly expressed in approximately 80% of EOC. Additionally, the ability of FRα to internalize relatively large molecules renders it suitable for developing targeted therapies (8, 9). Despite their anti-tumor effects in preclinical models, folate-cytotoxic drug conjugates and no conjugated humanized antibody have yet to demonstrate clinical efficacies (10). In contrast, mirvetuximab soravtansine (MIRV), or Elahere (ImmunoGen), the first FRα-targeting antibody-drug conjugate (ADC), has recently been approved by the US FDA to treat platinum-resistant ovarian cancer (11). Here, we summarize the biology of folate receptors, review different strategies to target FRα, and discuss potential mechanisms of ocular adverse events associated with MIRV. The approval of MIRV has renewed interest to develop other FRα-targeting therapeutics for treatment beyond EOC.

## 2 Folate transporter proteins

**Figure 1:**
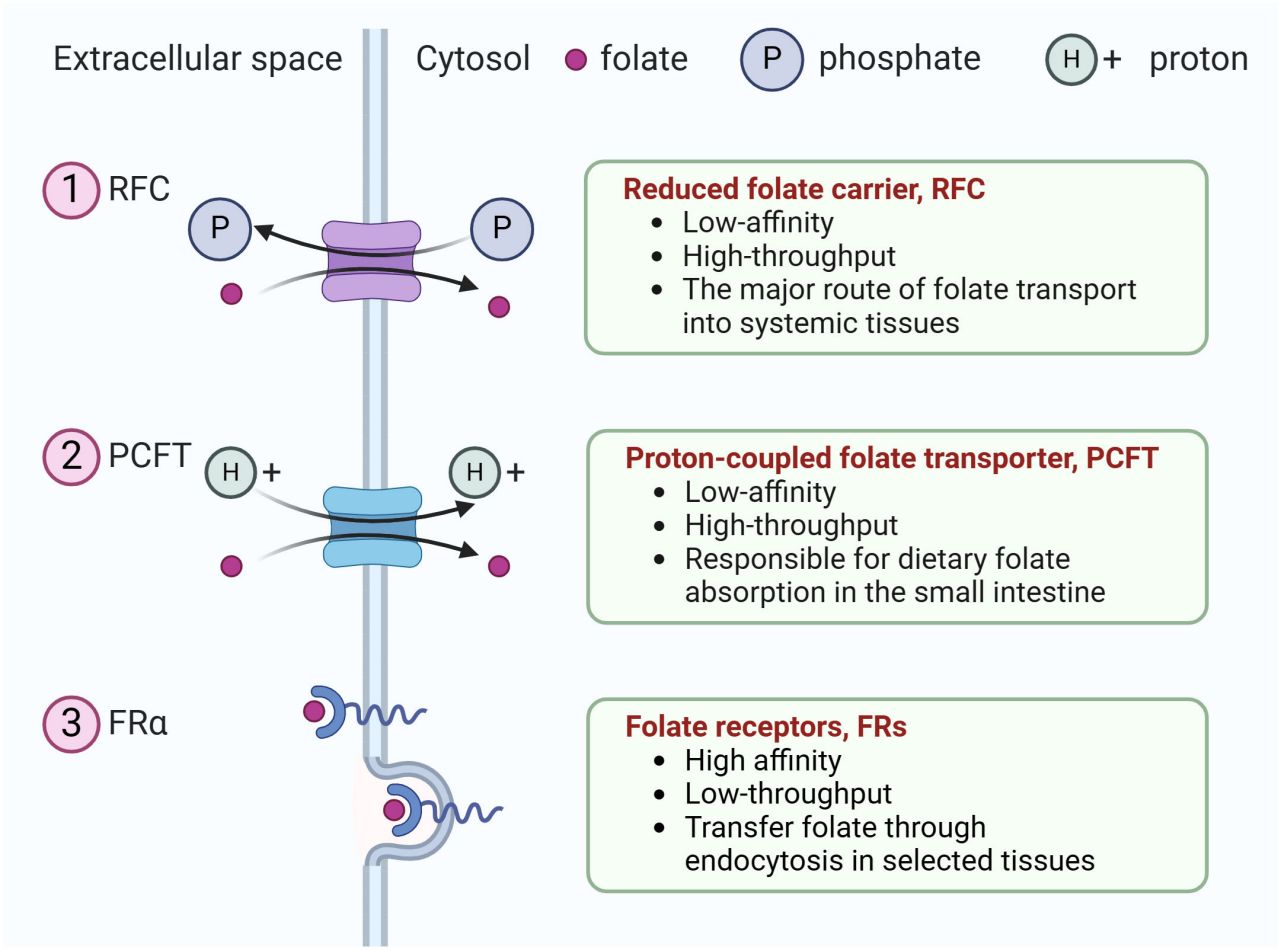
The three types of folate transporters. The uptake of extracellular folate is achieved mainly through three types of folate transporters. (1) RFC, an anion antiporter that uses a gradient of higher organic phosphate in the cell to transport folate into the cell while transporting organic phosphate out of the cell, (2) PCFT, a proton-coupled transporter, (3) folate receptor family (only FRα is shown). They transfer folate through endocytosis in selected tissues.

Humans cannot synthesize folate, an essential vitamin for eukaryotic cell proliferation and differentiation, and must obtain folate from dietary sources (12). The uptake of extracellular folate is achieved mainly through three types of folate transporters, including the reduced folate carrier, RFC (encoded by the SLC19A1 gene), the proton-coupled folate transporter, PCFT(encoded by the SLC46A1 gene), and folate receptors (FRs) (13). Ubiquitously expressed RFC serves as the major route of folate transport into systemic tissues (12), whereas PCFT is a proton-coupled transporter responsible for dietary folate absorption in the small intestine (14). Both RFC and PCFT are low-affinity, high-throughput transporters. In contrast, FRs are high affinity, low-throughput transporters that transfer folate through endocytosis in selected tissues (**Figure 1**).

Folate trafficking via FRα is considered to proceed via potocytosis, a lipid raft-mediated endocytosis mechanism (15). Folate binds specifically to FRα, forming a receptor-ligand complex, and subsequently intracellular vesicles are generated by invagination and budding off. Once internalized, the vesicles join together to from early endosomes, which acidify and fuse with lysosomes to release folates for the one-carbon metabolic reaction (16, 17).

There are four members in FRs family, including FRα (257aa, 30kDa), FRβ (255aa, 29kDa), FRγ (245aa, 28kDa) and FRδ (250aa, 28.6kDa), encoded by FOLR1 (Gene ID: 2348), FOLR2 (Gene ID: 2350), FOLR3 (Gene ID: 2352) and FOLR4 (Gene ID: 390243), respectively. FRs, also known as the folate binding proteins (FBPs), bind folic acid (FA) and 5-mTHF as well as folate-conjugated compounds with high affinity, and transport them inside cells by receptor-mediated endocytosis. FRα, FRβ and FRδ are all glycophosphatidylinositol (GPI) anchored cell-membrane proteins, whereas FRγ is a secreted protein lack of a GPI anchored region (18). FRα is the most studied family member, and is the focus of this Review. FRβ is mainly expressed in placental and myeloid leukocytes, including activated macrophages, tumor-infiltrating macrophages and acute as well as chronic myelogenous leukemia (19–21). FRβ-null mice are apparently normal, indicating that its function is dispensable to maintain organismal homeostasis (22). FRγ is expressed in neutrophil granulocytes and monocytes. FRδ, also named JUNO, is highly expressed in regulatory T cells and mammalian eggs. FRδ lacks the folate-binding pocket, and is unable to bind folate (23). The interaction between FRδ on the egg surface and IZUMO1 on the sperm surface is critical for mammalian fertilization as FRδ knockout eggs are unable to fuse with sperm (24).

FRα is mainly expressed on the plasma membrane of epithelial cells in several tissues, in particular the apical brush-border membrane of proximal renal tubular cells, retinal pigment epithelium, the choroid plexus (25), type1 and 2 pneumocytes in the lung, ovary, fallopian tube, uterus, cervix, epididymis, submandibular salivary gland, bronchial glands and trophoblasts in the placenta (26). FRα has a high affinity for reduced folates, such as tetrahydrofolate (THF), 5-mTHF and FA.

## 3 The role of FRα in health

FA is a nutrient essential for embryonic development. Folate deficiency can cause embryonic lethality with neural tube defects and orofacial anomalies (27, 28). FRα and its cargo FA are essential for proper mammalian embryogenesis. Knockout of the Folr1 gene is embryonic lethal in mice around the time of neural tube closure (22). Reduced FRα expression and function is associated with craniofacial anomalies, abnormal heart development, and neural tube defects (29). Consistently, daily maternal folate supplementation, before and during pregnancy markedly decreased embryonic mortality. Hundreds of genes were differentially expressed at the gestational day 9.5 between Folr1^-/-^ and wild-type embryos. These genes are implicated in the regulation of digestive and cardiovascular system development (27). In the placenta, FRα transports folates from the mother to the fetus (30, 31). Folate deficiency in pregnancy is associated with neural tube defects, restricted fetal growth and fetal programming of diseases later in life (32–34). Importantly, the risk of abnormal pregnancy outcomes is increased in pregnant women taking folate antagonists to treat cancer and other diseases.

FRα is also required to maintains functionalities of several organs in adult animal. Adult mice lacking Folr1 had lower blood folate levels and higher renal folate clearance rate (35). This is because kidneys maintain folate homeostasis in the body through glomerular filtration and tubular reabsorption process. The primary transporter for folate reabsorption in the kidneys is FRα, expressed on the apical surface of proximal tubular cells. FRα transports folate from the tubule lumens into tubular cells via receptor-mediated endocytosis (36). Kidney ischemia-reperfusion injury significantly reduces the expression of FRα and RFC, contributing to low folate level in acute kidney injury (AKI) (37). In spontaneously hypertensive rat (SHR), a deletion variant in the Folr1 promoter region results in impaired folate reabsorption in the renal tubules, and increased risk for diabetes mellitus and cardiovascular disease (38). Within the brain, FRα is selectively expressed in the choroid plexus, and promotes a vesicular transport of 5-mTHF across the choroid plexus (39). It has been reported that mutations in the FOLR1 gene cause cerebral folate transport deficiency resulting in a childhood onset neurodegenerative disease (40–42).

## 4 FRα in ovarian cancer

FRα is normally expressed in fallopian tube but not the ovary, consistent with EOC originating from the fallopian tube fimbriae rather than from ovary epithelial cells (20, 43). The expression of FRα can be regulated by folate levels. Folate deficiency increases FRα expression *in vivo* and *in vitro (* 44). Intracellular folate deficiency is associated with increased homocysteine. Homocysteine can promote the binding of heterogeneous nuclear ribonucleoprotein E1 (hnRNP E1) to the 5’ end of FOLR1 mRNA, upregulating FOLR1 expression at the level of translation (45). Folate deficiency also decreases DNA methylation, and global DNA hypomethylation may account for elevated of FRα expression in highly aggressive EOC (46). FRα levels correlate with histological stage and grade (47). A soluble form of FRα, known as soluble folate receptor (sFR), outperforms CA125 as a EOC recurrence marker, even when the CA125 level remains low (18, 48, 49).

### 4.1 FRα as transporter

It has been proposed that FRα promotes tumorigenesis by increasing folates for one-carbon metabolism (50). However, even when FRα is overexpressed, the main route to transport folate into cells is RFC. RFC accounts for 70% of the uptake of the serum folate 5-mTHF (51). Thus, it is unlikely that increasing folate levels is the primary mechanism of FRα to promote tumorigenesis.

### 4.2 FRα as transcription factor

Once entering cells by endocytosis, FRα and associated FA can activate several cellular pathways. FRα can translocate into the nucleus and function as a transcription factor to promote the expression of several genes including Oct4, Sox2, Klf4 (52), Hes1 and Fgfr4 (53).

### 4.3 FRα and cell signaling

In addition, FA, together with FRα, can interact with gp130 to initiate the JAK-STAT3 pathway. Phosphorylated -STAT3 transcriptionally activates its target genes frequently associated with unfavorable patient outcomes (54, 55). The FRα-FA complex also physically interacts with progesterone receptor to promote ERK1/2 phosphorylation (56). FRα can also promote cancer cell metastasis by downregulating the intercellular adhesion molecule E-cadherin (51, 57).

## 5 Therapeutic strategies targeting FRα

**Figure 2:**
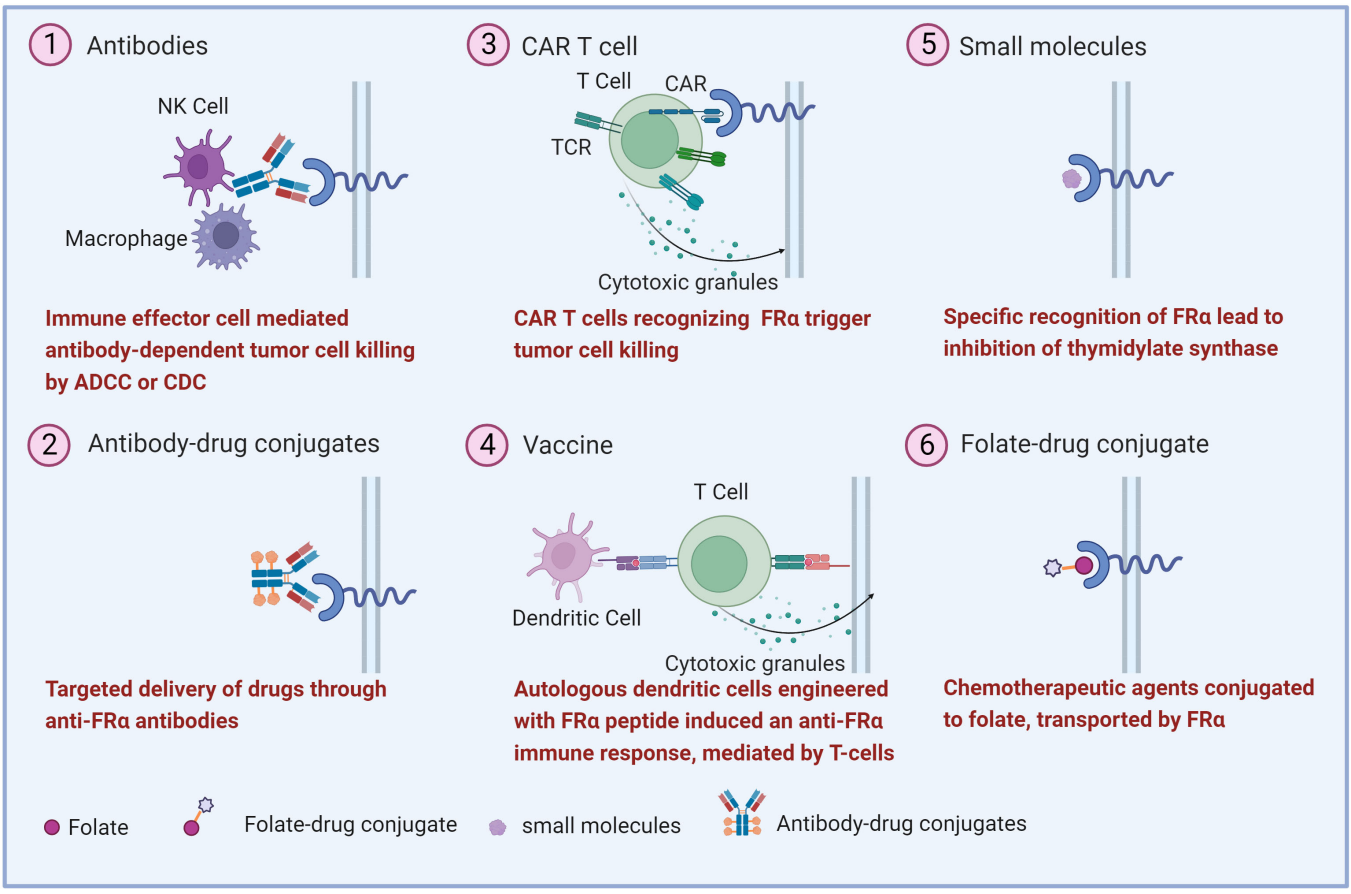
Overview of the therapeutic strategies targeting FRα. Various FRα-target strategies in ovarian cancer have been explored including (1) monoclonal antibodies, (2) antibody-drug conjugates, (3) Chimeric antigen receptor (CAR) T cell, (4) vaccine, (5) small molecule and, (6) folate-drug conjugate.

The high expression of FRα in malignant tumors makes it a potential target for anti-tumors drug development. Various strategies have been explored, including monoclonal antibodies, antibody-drug conjugate (ADC), FRα-specific CAR T, vaccines, small molecules, and folate-drug conjugate (**Figure 2**) (17, 58). Several clinical trials involving FRα-targeted agents are currently ongoing (**Table 1**). Notably, an ADC drug has recently been approved by US FDA.

**Table 1:** Key clinical trials using FRα-targeting agents to treat ovarian cancer.

Compound/Drug	Mechanism	Clinical trial	Outcome	Refs
Monoclonal antibodies				
**Farletuzumab/MORab003**	ADCC and CDC	Phase I: Epithelial ovarian, fallopian, or primary peritoneal carcinoma (n=25)	Safe and well tolerated	(59)
		Phase II: relapsed platinum-sensitive ovarian cancer (n=54)	Enhance the response rate and duration of response in recurrent, platinum-sensitive ovarian cancer patients	(60)
		Phase III: ovarian cancer in first platinum-sensitive relapse (n=1100)	Failed to reach PFS endpoints	(61)
**MOv18 (IgE)**	ADCC and CDC	Phase I: solid tumors expressing FRα (n=26), NCT02546921	Safe and promising antitumor activity in FRα-positive solid tumors	(62)
Antibody-drug conjugate				
**MORAB-202**	Targeted delivery of drugs through anti- FRα antibodies	Phase I: FRα-positive advanced solid tumors (n=22) NCT03386942	Well-tolerated and promising antitumor activity in FRα-positive solid tumors	(63)
**Mirvetuximab Soravtansine/ MIRV/Elahere/IMGN853**	Targeted delivery of drugs through anti-FRα antibodies	Phase I: FRα-positive solid tumors include ovarian cancer (n=44), NCT01609556	Safe and encouraging efficacy	(64)
		Phase Ib: patients with platinum-resistant epithelial ovarian, fallopian tube, or primary peritoneal cancer (n=66), NCT02606305	The combination of MIRV with bevacizumab is well tolerated in patients with platinum-resistant, recurrent ovarian cancer	(65)
		Phase II: platinum-resistant epithelial ovarian cancer (PROC) (n=106), NCT04296890	Favorable tolerability, safety and encouraging efficacy in patients with FRα-high PROC who had received up to three prior therapies	(66)
		Phase III: FRα-positive platinum-resistant ovarian cancer (n=366), NCT02631876	Primary endpoint PFS was not reached	(67)
CAR-T				
**Anti- FRα CAR-T+IL-2**	CAR-T cells recognizing FRα	Phase I: ovarian cancer (n=14)	Not effective, likely due to short-term survival of CAR-T cells	(68)
Vaccine				
**E39+GM-CSF**	Cytotoxic T cell reponse elicited by a FRα-dervied peptide	Phase I/IIa: ovarian and endometrial cancer (n=51)	Safe and encouraging efficacy	(69)
**Multi-epitope FRα peptide**	Cytotoxic T cell response elicited by 5 FRα-derived peptides	Phase I: Ovarian cancer and breast cancer (n=22), NCT01606241	Safe and encouraging efficacy	(70)
Small molecules				
**BGC945/CT900/ONX-0801**	Thymidylate synthase inhibitor transported via FRα into cancer cells	Phase I: High-grade serous ovarian cancer (n=109) NCT02360345	Acceptable side effect profiles and significant clinical activity	(71)
Folate-drug conjugate				
**EC145/Vintafolide**	Chemotherapeutic agents conjugated to folate, transported by FRα	Phase I: refractory solid tumors include ovarian cancer (n=32), NCT00308269	partial response	(72)
		Phase II: recurrent platinum-resistant ovarian cancer who had undergone no more than two prior cytotoxic regimens (n=162), NCT00722592	EC145 plus PLD is superior to the standard therapy	(73)

### 5.1 Antibodies

Several FRα-targeting antibodies have been developed, including farletuzumab (IgG1) (74), MOv18 (IgG1) (75), MOv18 (IgE) (76) and MOv19 (IgG2A). Farletuzumab (MORab003; Morphotek, Inc.), the first anti-FRα monoclonal antibody, exhibited anti-tumor activities potentially via inducing antibody-dependent cellular cytotoxicity (ADCC), complement-dependent cytotoxicity (CDC), and persistent tumor cell autophagy leading to reduced cell proliferation and inhibition of the Lyn kinase signaling pathway (77). In a phase I study, farletuzumab showed negligible toxicity in patients with EOC (59). In the phase II study, farletuzumab with carboplatin and taxane enhanced the response rate and duration of response in platinum-sensitive ovarian cancer patients (60). Unfortunately, PFS was not reached in the phase III clinical trial in ovarian cancer patients (61). Nevertheless, farletuzumab was adopted to be the anti-FRα component in ADC drug MORAb-202. MOv18 (IgG1) was not further developed. In a phase I study (NCT02546921), MOv18 (IgE), a chimeric first-in-class IgE antibody, exhibits anti-tumor effectiveness in ovarian cancer patients, with transient urticaria being the most frequent side effect (62). MOv19 (IgG2A) was developed in 1980s. Since then, two derivatives of MOv19 have entered the clinical trials. One is M9346A (78), and the other is chimeric antigen receptor (CAR) composed of a MOv19 anti-FRα specific single chain variable fragment (79). M9346A is the anti-FRα antibody component of MIRV (80).

### 5.2 Anti-FRα ADC

ADC is a drug delivery system, composed of a tumor-targeting monoclonal antibody and a cytotoxic payload joined by a linker (81). Conceptually, this configuration of ADC facilitates the delivery of cytotoxic drugs specifically to tumor cells, and thus should minimize the damage to normal tissues. However, due to the high affinity of antibody-antigen interaction, ADC could target normal tissues expressing a low level of antigen. Thus, the toxicity profile of ADC may be different from unconjugated cytotoxic payload (1). The innate ability of FRα to internalize large molecules makes it a suitable target for delivering ADC.

#### 5.2.1 MORAb-202

MORAb-202, an ADC that combines the humanized anti-human FRα antibody farletuzumab with the microtubule-targeting drug eribulin, has demonstrated substantial anticancer efficacy in cancer cell lines and in patient-derived xenograft models (63, 82). Of note, eribulin is a license drug to treat metastatic breast cancer in the United States (83, 84). In contrast, payloads in other ADCs are too toxic to be used alone. MORAb-202 is anticipated to cause immunogenic cell death, as has been shown with previous tubulin inhibitor-based ADCs such as T-DM1 (85). The toxicity and pharmacokinetics of MORAb-202 were studied in a cynomolgus monkey model at various dosages (83). The bone marrow was the primary target of MORAb-202 toxicity in monkeys, mostly due to the payload eribulin (86). The efficacy of MORAb-202 depends on the expression level of FRα both *in vitro* and *in vivo (* 87). MORAb-202 is now undergoing phase I/II clinical trials to assess its effect in FRα-positive solid tumors (63, 82).

#### 5.2.2 Mirvetuximab soravtansine

MIRV (IMGN853, Elahere) developed by ImmunoGen, is the first ADC to target FRα-expressing tumor cells. It consists of a humanized anti-FRα monoclonal antibody (M9346A) (88), a cleavable linker sulfo-SPDB, and the cytotoxic maytansionoid effector molecule DM4 (88). Once DM4 is accumulated intracellularly, it acts as a potent antimitotic agent by suppressing microtubule dynamics (89). In 2022, MIRV received accelerated approval by US FDA for the treatment of adult patients with FRα-positive, platinum-resistant epithelial ovarian cancer (PROC), fallopian tube cancer or primary peritoneal cancer, previously treated with 1-3 prior systemic anti-cancer regimens (11).

MIRV is taken up by tumor cells through antigen-mediated endocytosis, transported to lysosomes by vesicular trafficking, and degraded to release lysine-Nϵ-sulfo-SPDB-DM4. The lysine-DM4 is further reduced and S-methylated within the cell, generating hydrophobic maytansinoid derivatives, DM4 and S-methyl-DM4. These three catabolites can inhibit tubulin polymerization and microtubule assembly, leading to cell death. Furthermore, DM4 and S-mehtyl-DM4 can diffuse into intercellular space to kill bystander cells (90). An expansion cohort study of the phase I trial (NCT01609556) found that FRα expression remained stable in biopsy samples following two doses of MIRV, although reductions in post-treatment levels were seen in some patients (91).

The efficacy of MIRV against epithelial ovarian cancer has been investigated in several clinical trials as monotherapy or in combination with other anti-tumor drugs (92). The first-in-human, phase I study (NCT01609556) of MIRV as single agent in patient with EOC and other FRα-positive solid tumors has provided preliminary data on safety and efficacy. A total of 44 patients were enrolled, and the strongest clinical benefit was observed in two EOC patients (64). Thus, additional cohorts were extended as part of the same trial to include individuals with advanced EOC, primary peritoneal or fallopian tube cancers. The objective response rate (ORR) was 22%, and a superior efficacy was observed in the subset of patients with the highest FRα levels (ORR, 31%, PFS 5.4 months) (91). The positive association between FRα expression levels and the efficacy of MIRV prompted another phase I trial, consisting of 46 patients with strong FRα expression (defined as ≥25% of cells with at least 2+ staining intensity by immunohistochemistry). The ORR was 26% and median PFS was 4.8 months (93). These studies established that MIRV had a manageable safety profile, and was effective to control FRα-positive PROC.

In-depth analysis of the phase I results indicated that the response rate was correlated with the number of prior therapies. Patients received four or more priors had a lower response rate (ORR, 13%; PFS 3.9 months) compared with ones received one to three priors (93). On the basis of this observation, the first randomized, multicenter phase III study, FORWARD I (NCT02631876), enrolled platinum-resistant patients (FRα-positive PROC, primary peritoneal or fallopian tube cancer) who have received one to three prior therapies and with high or medium levels of FRα expression, defined as staining intensity ≥2+ in>75% or 50-74% cells, respectively (94). The purpose of this study was to compare the safety and efficacy of MIRV with chemotherapies of investigator’s choice (94). A total of 113 ovarian cancer patients were randomly assigned to receive MIRV or chemotherapies of investigator’s choice (36 patients in the MIRV arm). The efficacy of the MIRV arm (ORR, 47%; PFS 6.7 months) was superior to outcomes typically seen with established single-agent chemotherapy, including paclitaxel, pegylated liposomal doxorubicin and topotecan. This encouraging result prompted another phase III FORWARD I trial with an expanded population. 366 platinum-resistant ovarian cancer patients were randomly assigned to receive MIRV or chemotherapies of investigator’s choice in a 2:1 ratio. However, MIRV did not result in a significant improvement in PFS compared with standard chemotherapy (67), demonstrating that the efficacy of MIRV as monotherapy is limited.

Subsequent clinical trials explored combinatorial approaches. Preclinical studies indicate that MIRV can synergize with carboplatin, doxorubicin, bevacizumab and pegylated liposomal doxorubicin to kill ovarian cancer cells *in vitro* and *in vivo (* 95). In FORWARD II trials (65, 96), patients with FRα positive PROC were treated with MIRV and bevacizumab. The objective response rate (ORR) was 39%, including 5 complete responses and 21 partial responses. The median PFS was 6.9 months (65). Thus, the combination of MIRV plus bevacizumab is effective, with long-lasting responses and a manageable safety profile in patients with PROC. A single-arm, phase II study, SORAY (NCT04296890) enrolled 106 FRα-high PROC patients previously undergone one to three treatments, including bevacizumab (66). ORR was 32.4%, with 5 complete and 29 partial responses. The ORR by investigator was 35.3% in patients with one to two priors and 30.2% in patients with three priors. Interestingly, the ORR by investigator was 38% in patients with prior PARP inhibitor exposure and 27.5% in those without (66).

##### 5.2.2.1 MIRV treatment-related ocular adverse effects

ADCs are expected to target tumor cells with high specificity, and are less toxicity to normal cells than conventional chemotherapies. However, most ADCs exhibit similar toxicity profiles with their cytotoxic payloads (97). The most common treatment-related adverse effects of MIRV were diarrhea, blurred vision, nausea, and fatigue. Most of these adverse events were mild (grade 1 or 2) and were readily manageable with supportive care (64, 91, 93, 97). Reversible ocular adverse events (AEs), primarily corneal keratopathy and blurred vision, frequently occurred among patients (98). This ocular toxicity is likely caused by DM4, as it has been observed in patients treated with other antibody-DM4 conjugates (99, 100). The underlying cause of ocular toxicity is not clear. FRα expression is negative in the eye based on immunohistochemistry. However, the expression of FRα has not been formally ruled out by more sophisticated techniques such as single-cell sequencing. The preventive use of topical corticosteroid eye drops can reduce but not eliminate ocular AEs (101, 102). Further mechanistic studies will be required to disentangle the underlying causes.

### 5.3 FRα-specific CAR-T

Preclinical investigations have indicated that FRα-specific chimeric antigen receptor (CAR) T cell therapy has promising antitumor effects (103, 104). A phase I trial of a FRα-specific CAR T cell therapy in patients with ovarian cancer showed no reduction in tumor burden, because these T cells did not survive well (68). The addition of costimulatory signals, including CD27, CD28, CD134 (OX-40) and CD137 (4-1BB) into CARs have been shown to promote T-cell survival (104, 105). An improved strategy engineering FRα-specific CAR with a CD137 costimulatory signaling domain in tandem enhanced T-cell persistence in tumor bed, but antitumor activity was still minimal (106). A novel Tandem-CAR encoding an anti-FRα scFv, an anti-MSLN scFv, and two peptide sequences of IL-12 were designed to improve the efficacy, infiltration, persistence, and proliferation of CAR-T cell in ovarian cancer (107). Furthermore, CAR T cells, composed of MOv19 anti-FRα-specific single chain variable fragment fused to 4-1BB and TCRzeta signaling domains (MOv19-BBZ), is currently evaluated by a phase I clinical trial in recurrent high grade serous ovarian cancer patients (78).

### 5.4 Vaccines

Peptide-based vaccine is another strategy to stimulate antitumor immunity (108, 109). FRα-derived peptides E39 (amino acid 191-199) and E41 (amino acid 245-253) were shown to be immunogenic (110). In a phase I/IIa trial with 51 patients, E39 plus GM-CSF was safe and might be beneficial in preventing the recurrence of high-risk ovarian and endometrial cancers (69). In another phase I clinical trial, the safety and immunogenicity of five FRα-derived peptides were examined in breast and ovarian cancer patients (70). These studies demonstrate that FRα-derived peptides are safe, but their clinical efficacy awaits further investigation.

### 5.5 Other approaches

#### 5.5.1 Small molecule

BGC 945 (also known as ONX-0801 or CT900) is a thymidylate synthase inhibitor internalized by FRα (111). In a recent phase I clinical trial, the most common BGC945 treatment-related adverse events were fatigue, nausea, diarrhea, cough, anemia, and pneumonitis. Clinical benefit was seen in high-grade serous ovarian cancer patients with medium to high FRα expression (71).

#### 5.5.2 Folate-drug conjugate

It is reasonable to assume that folate-based drug conjugates can enter FRα-expressing cells via endocytosis. The drug conjugates will subsequently be released from FRα due to acidic environment in endosomes, and accumulate intracellularly.

##### 5.5.2.1 Preclinical reagents

EC131, the first folate-drug conjugate, consists of a potent microtubule-stabilizing agent, DM1, linked to FA by intramolecular disulfide bonds. EC131 has not been tested clinically. EC2629 is a folate conjugate of a DNA crosslinking agent pyrrolobenzodiazepine (PBD) linked by a novel DNA-alkylating moiety. Preclinical studies demonstrate that EC2629 has antitumor activity in ovarian, endometrial, and triple negative breast cancers (112). Notably, most ADCs using PBD as the payload are now halted due to excessive toxicity of PBD. No literature regarding EC2629 had been published since 2020, suggesting that its development may be halted as well. BMS753493 is a folate conjugate of the epothilone analog. The frequency and severity of peripheral neuropathy and neutropenia was less in patients treated with BMS748285 than epothilones. However, little efficacy was observed in solid tumors including ovarian cancer, and further development of BMS753493 was halted (113).

##### 5.5.2.2 Agents in clinical stage

EC145 (vintafolide) is a water-soluble derivative of FA linked to the vinca alkaloid desacetylvinblastine hydrazide (DAVLBH). In a phase I clinical trial, one partial response was observed in a patient with metastatic ovarian cancer (72). In a randomized phase II trial of patients with platinum-resistant ovarian cancer, EC145 plus pegylated liposomal doxorubicin exhibited efficacy superior to the standard therapy (73). Unfortunately, in the phase III clinical trial (NCT01170650), the PFS in ovarian cancer patients was not reached (114).

## 6 Conclusion and future perspectives

The understanding of the molecular characteristics of EOC have advanced in the past decade. However, platinum resistance remains a major clinical challenge, and renders EOC the most fatal gynecological malignancy. Angiogenesis inhibitors (bevacizumab) and PARP inhibitors (olaparib, rucaparib, and niraparib) have not significantly increased overall survival in most patients. Innovative and effective therapeutic strategies are urgently needed. In this regard, FRα has emerged as an appealing and clinically verified candidate for the development of targeted therapies. The relatively enriched expression of FRα on the surface of cancer cells and the ability of FRα to transport cytotoxic payloads into cancer cells have inspired the development of various therapeutic modalities including antibodies, ADCs, CAR T, vaccines, small molecules, and folate-drug conjugate. Notably, MIRV, a FRα-targeting ADC, has recently been approved by US FDA to treat adult patients with PROC, fallopian tube cancer or primary peritoneal cancer. Several promising FRα-targeting modalities are under clinical evaluation. It will be of interest to see their efficacy on EOC and other FRα-expressing cancer types.

It is also interesting that ADC is the only FRα-targeting modality that has achieved clinical efficacy so far. We speculate that the inhibition of FRα function via monoclonal antibodies may not be enough to inhibit tumor growth. This is because FRα is not a major survival signaling pathway even in FRα-high tumors. In addition, RFC is the major folate transporter and co-expressed with FRα. Although folate is an essential vitamin, suppressing FRα activity is not sufficient to block folate transport into cells. On the other hand, folate-drug conjugates can act similarly as FRα-targeting ADCs to deliver toxic payload into FRα-high cells. However, considering that RFC and PCFT are major folate transporters in many tissues, folate-drug conjugates likely can enter any cells expressing RFC and PCFT. Thus, folate-drug conjugates likely have less targeting specificity and therapeutical index than FRα-targeting ADCs.

In our opinion, further basic and clinical investigations are warranted to maximize the clinical efficacy of MIRV. MIRV is currently only approved for ovarian cancers with high expression of FRα. Considering that FRα is highly expressed in several cancer types, MIRV may be effective in these contexts. In addition, MIRV is known for its bystander effect. Therefore, MIRV may benefit patients with cancers expressing low to moderate level of FRα, analogous to the situation of HER2-targeting ADC, DS-8201a. Lastly, blurred vision occurs in 50-60% of patients treated with MIRV (115, 116). This peculiar high prevalence of ocular toxicity is uncommon in other ADCs, and can be debilitating for patients in our experience. The exact pathological mechanism is yet to be elucidated to improve the prophylactic treatment. Undoubtedly, the landmark approval of MIRV will fuel the interest to develop novel FRα-targeting diagnostic and therapeutic approaches to treat cancer.

## Author contributions

JM collected the related paper and drafted the manuscript. LW and LY created the figures. TS, XL, RY, YJ and JL revised this manuscript. QL conceived the structure of manuscript and revised the manuscript. All authors read and approved the final manuscript. All authors contributed to the article and approved the submitted version.

## References

[1] Calo CA O'Malley DM. Antibody-drug conjugates for the treatment of ovarian cancer. Expert Opin Biol Ther (2021) 21(7):875–87. doi: 10.1080/14712598.2020.1776253b 32463296

[2] Kuroki L Guntupalli SR. Treatment of epithelial ovarian cancer. BMJ (2020) 371:m3773. 10.1136/bmj.m3773 33168565 10.1136/bmj.m3773

[3] Yang L Xie HJ Li YY Wang X Liu XX Mai J. Molecular mechanisms of platinum−based chemotherapy resistance in ovarian cancer (Review). Oncol Rep (2022) 47(4):82. doi: 10.3892/or.2022.8293 35211759 PMC8908330

[4] Zamarin D. Novel therapeutics: response and resistance in ovarian cancer. Int J Gynecol Cancer (2019) 29(Suppl 2):s16–21. doi: 10.1136/ijgc-2019-000456 PMC7368996 31462544

[5] Arend RC Jackson-Fisher A Jacobs IA Chou J Monk BJ. Ovarian cancer: new strategies and emerging targets for the treatment of patients with advanced disease. Cancer Biol Ther (2021) 22(2):89–105. doi: 10.1080/15384047.2020.1868937 33427569 PMC7928025

[6] Siegel RL Miller KD Wagle NS Jemal A. Cancer statistics, 2023. CA Cancer J Clin (2023) 73(1):17–48. doi: 10.3322/caac.21763 36633525

[7] Siegel RL Miller KD Fuchs HE Jemal A. Cancer statistics, 2022. CA Cancer J Clin (2022) 72(1):7–33. doi: 10.3322/caac.21708 35020204

[8] Walters CL Arend RC Armstrong DK Naumann RW Alvarez RD. Folate and folate receptor alpha antagonists mechanism of action in ovarian cancer. Gynecol Oncol (2013) 131(2):493–8. doi: 10.1016/j.ygyno.2013.07.080 23863359

[9] Scaranti M Cojocaru E Banerjee S Banerji U. Exploiting the folate receptor α in oncology. Nat Rev Clin Oncol (2020) 17(6):349–59. doi: 10.1038/s41571-020-0339-5 32152484

[10] Bergamini A Ferrero S Leone Roberti Maggiore U Scala C Pella F Vellone VG. Folate receptor alpha antagonists in preclinical and early stage clinical development for the treatment of epithelial ovarian cancer. Expert Opin Investigat Drugs (2016) 25(12):1405–12. doi: 10.1080/13543784.2016.1254616 27797594

[11] Heo YA. Mirvetuximab soravtansine: first approval. Drugs (2023) 83(3):265–73. doi: 10.1007/s40265-023-01834-3 36656533

[12] Zhao R Goldman ID. Folate and thiamine transporters mediated by facilitative carriers (SLC19A1-3 and SLC46A1) and folate receptors. Mol Aspects Med (2013) 34(2-3):373–85. doi: 10.1016/j.mam.2012.07.006 PMC3831518 23506878

[13] Zhao R Diop-Bove N Visentin M Goldman ID. Mechanisms of membrane transport of folates into cells and across epithelia. Annu Rev Nutr (2011) 31:177–201. doi: 10.1146/annurev-nutr-072610-145133 21568705 PMC3885234

[14] Visentin M Diop-Bove N Zhao R Goldman ID. The intestinal absorption of folates. Annu Rev Physiol (2014) 76:251–74. doi: 10.1146/annurev-physiol-020911-153251 PMC3982215 24512081

[15] Sabharanjak S Mayor S. Folate receptor endocytosis and trafficking. Adv Drug Deliv Rev (2004) 56(8):1099–109. doi: 10.1016/j.addr.2004.01.010 15094209

[16] Menezo Y Elder K Clement A Clement P. Folic acid, folinic acid, 5 methyl tetraHydroFolate supplementation for mutations that affect epigenesis through the folate and one-carbon cycles. Biomolecules (2022) 12(2):197. doi: 10.3390/biom12020197 35204698 PMC8961567

[17] Cheung A Bax HJ Josephs DH Ilieva KM Pellizzari G Opzoomer J. Targeting folate receptor alpha for cancer treatment. Oncotarget (2016) 7(32):52553–74. doi: 10.18632/oncotarget.9651 PMC5239573 27248175

[18] Holm J Hansen SI. Characterization of soluble folate receptors (folate binding proteins) in humans. Biological roles and clinical potentials in infection and Malignancy. Biochim Biophys Acta Proteins Proteomics (2020) 1868(10):140466. doi: 10.1016/j.bbapap.2020.140466 32526472

[19] Puig-Kröger A Sierra-Filardi E Domínguez-Soto A Samaniego R Corcuera MT Gómez-Aguado F. Folate receptor beta is expressed by tumor-associated macrophages and constitutes a marker for M2 anti-inflammatory/regulatory macrophages. Cancer Res (2009) 69(24):9395–403. doi: 10.1158/0008-5472.Can-09-2050 19951991

[20] O'Shannessy DJ Somers EB Wang LC Wang H Hsu R. Expression of folate receptors alpha and beta in normal and cancerous gynecologic tissues: correlation of expression of the beta isoform with macrophage markers. J Ovarian Res (2015) 8:29. doi: 10.1186/s13048-015-0156-0 25971554 PMC4464638

[21] Han W Zaynagetdinov R Yull FE Polosukhin VV Gleaves LA Tanjore H. Molecular imaging of folate receptor β-positive macrophages during acute lung inflammation. Am J Respir Cell Mol Biol (2015) 53(1):50–9. doi: 10.1165/rcmb.2014-0289OC PMC4566110 25375039

[22] Piedrahita JA Oetama B Bennett GD van Waes J Kamen BA Richardson J. Mice lacking the folic acid-binding protein Folbp1 are defective in early embryonic development. Nat Genet (1999) 23(2):228–32. doi: 10.1038/13861 10508523

[23] Bianchi E Doe B Goulding D Wright GJ. Juno is the egg Izumo receptor and is essential for mamMalian fertilization. Nature (2014) 508(7497):483–7. doi: 10.1038/nature13203 PMC3998876 24739963

[24] Kato K Satouh Y Nishimasu H Kurabayashi A Morita J Fujihara Y. Structural and functional insights into IZUMO1 recognition by JUNO in mamMalian fertilization. Nat Commun (2016) 7:12198. doi: 10.1038/ncomms12198 27416963 PMC4947182

[25] Olney KC Todd KT Pallegar PN Jensen TD Cadiz MP Gibson KA. Widespread choroid plexus contamination in sampling and profiling of brain tissue. Mol Psychiatry (2022) 27(3):1839–47. doi: 10.1038/s41380-021-01416-3 PMC9095494 34983929

[26] Elnakat H Ratnam M. Distribution, functionality and gene regulation of folate receptor isoforms: implications in targeted therapy. Adv Drug Deliv Rev (2004) 56(8):1067–84. doi: 10.1016/j.addr.2004.01.001 15094207

[27] Seelan RS Mukhopadhyay P Philipose J Greene RM Pisano MM. Gestational folate deficiency alters embryonic gene expression and cell function. Differentiation (2021) 117:1–15. doi: 10.1016/j.diff.2020.11.001 33302058 PMC7855679

[28] Alam C Kondo M O'Connor DL Bendayan R. Clinical implications of folate transport in the central nervous system. Trends Pharmacol Sci (2020) 41(5):349–61. doi: 10.1016/j.tips.2020.02.004 32200980

[29] Rosenquist TH Chaudoin T Finnell RH Bennett GD. High-affinity folate receptor in cardiac neural crest migration: a gene knockdown model using siRNA. Dev Dynamics (2010) 239(4):1136–44. doi: 10.1002/dvdy.22270 20235221

[30] Caviedes L Iniguez G Hidalgo P Castro JJ Castano E Llanos M. Relationship between folate transporters expression in human placentas at term and birth weights. Placenta (2016) 38:24–8. doi: 10.1016/j.placenta.2015.12.007 26907378

[31] Jessel RH Rosario FJ Chen YY Erickson K Teal SB Kramer A. Decreased placental folate transporter expression and activity in first and second trimester in obese mothers. J Nutr Biochem (2020) 77:108305. doi: 10.1016/j.jnutbio.2019.108305 31926453

[32] Chen YY Gupta MB Grattton R Powell TL Jansson T. Down-regulation of placental folate transporters in intrauterine growth restriction. J Nutr Biochem (2018) 59:136–41. doi: 10.1016/j.jnutbio.2018.06.003 PMC6129407 29986308

[33] Ramaekers VT Quadros EV. Cerebral folate deficiency syndrome: early diagnosis, intervention and treatment strategies. Nutrients (2022) 14(15):3096. doi: 10.3390/nu14153096 35956272 PMC9370123

[34] Piñuñuri R Castaño-Moreno E Llanos MN Ronco AM. Epigenetic regulation of folate receptor-α (FOLR1) in human placenta of preterm newborns. Placenta (2020) 94:20–5. doi: 10.1016/j.placenta.2020.03.009 32421530

[35] Birn H Spiegelstein O Christensen EI Finnell RH. Renal tubular reabsorption of folate mediated by folate binding protein 1. J Am Soc Nephrol JASN (2005) 16(3):608–15. doi: 10.1681/asn.2004080711 15703271

[36] Shillingford JM Leamon CP Vlahov IR Weimbs T. Folate-conjugated rapamycin slows progression of polycystic kidney disease. J Am Soc Nephrol JASN (2012) 23(10):1674–81. doi: 10.1681/ASN.2012040367 PMC3458469 22859856

[37] Yang C Wijerathne CUB Tu GW Woo CWH Siow YL Madduma Hewage S. Ischemia-reperfusion injury reduces kidney folate transporter expression and plasma folate levels. Front Immunol (2021) 12:678914. doi: 10.3389/fimmu.2021.678914 34149715 PMC8213029

[38] Pravenec M Kozich V Krijt J Sokolova J Zidek V Landa V. Genetic variation in renal expression of folate receptor 1 (Folr1) gene predisposes spontaneously hypertensive rats to metabolic syndrome. Hypertension (2016) 67(2):335–41. doi: 10.1161/HYPERTENSIONAHA.115.06158 26667416

[39] Grapp M Wrede A Schweizer M Huwel S Galla HJ Snaidero N. Choroid plexus transcytosis and exosome shuttling deliver folate into brain parenchyma. Nat Commun (2013) 4:2123. doi: 10.1038/ncomms3123 23828504

[40] Grapp M Just IA Linnankivi T Wolf P Lucke T Hausler M. Molecular characterization of folate receptor 1 mutations delineates cerebral folate transport deficiency. Brain (2012) 135(Pt 7):2022–31. doi: 10.1093/brain/aws122 22586289

[41] Zhang C Deng X Wen Y He F Yin F Peng J. First case report of cerebral folate deficiency caused by a novel mutation of FOLR1 gene in a Chinese patient. BMC Med Genet (2020) 21(1):235. doi: 10.1186/s12881-020-01162-3 33243190 PMC7691102

[42] Han X Cao X Cabrera RM Pimienta Ramirez PA Zhang C Ramaekers VT. KDM6B variants may contribute to the pathophysiology of human cerebral folate deficiency. Biology (2022) 12(1):74. doi: 10.3390/biology12010074 36671766 PMC9855468

[43] O'Shannessy DJ Somers EB Smale R Fu YS. Expression of folate receptor-alpha (FRA) in gynecologic Malignancies and its relationship to the tumor type. Int J Gynecol Pathol (2013) 32(3):258–68. doi: 10.1097/PGP.0b013e3182774562 23518909

[44] Kelemen LE. The role of folate receptor alpha in cancer development, progression and treatment: cause, consequence or innocent bystander? Int J Cancer (2006) 119(2):243–50. doi: 10.1002/ijc.21712 16453285

[45] Antony A Tang YS Khan RA Biju MP Xiao X Li QJ. Translational upregulation of folate receptors is mediated by homocysteine via RNA-heterogeneous nuclear ribonucleoprotein E1 interactions. J Clin Invest (2004) 113(2):285–301. doi: 10.1172/jci11548 14722620 PMC310746

[46] Notaro S Reimer D Fiegl H Schmid G Wiedemair A Rossler J. Evaluation of folate receptor 1 (FOLR1) mRNA expression, its specific promoter methylation and global DNA hypomethylation in type I and type II ovarian cancers. BMC Cancer (2016) 16:589. doi: 10.1186/s12885-016-2637-y 27485273 PMC4971744

[47] Bax HJ Chauhan J Stavraka C Santaolalla A Osborn G Khiabany A. Folate receptor alpha in ovarian cancer tissue and patient serum is associated with disease burden and treatment outcomes. Br J Cancer (2023) 128(2):342–53. doi: 10.1038/s41416-022-02031-x PMC9902484 36402875

[48] Giampaolino P Foreste V Della Corte L Di Filippo C Iorio G Bifulco G. Role of biomarkers for early detection of ovarian cancer recurrence. Gland Surg (2020) 9(4):1102–11. doi: 10.21037/gs-20-544 PMC7475347 32953625

[49] Farran B Albayrak S Abrams J Tainsky MA Levin NK Morris R. Serum folate receptor α (sFR) in ovarian cancer diagnosis and surveillance. Cancer Med (2019) 8(3):920–7. doi: 10.1002/cam4.1944 PMC6434204 30761774

[50] Wallace-Povirk A Hou Z Nayeen MJ Gangjee A Matherly LH. Folate transport and one-carbon metabolism in targeted therapies of epithelial ovarian cancer. Cancers (2021) 14(1):191. doi: 10.3390/cancers14010191 35008360 PMC8750473

[51] Siu MK Kong DS Chan HY Wong ES Ip PP Jiang L. Paradoxical impact of two folate receptors, FRalpha and RFC, in ovarian cancer: effect on cell proliferation, invasion and clinical outcome. PloS One (2012) 7(11):e47201. doi: 10.1371/journal.pone.0047201 23144806 PMC3492371

[52] Mohanty V Shah A Allender E Siddiqui MR Monick S Ichi S. Folate Receptor Alpha Upregulates Oct4, Sox2 and Klf4 and Downregulates miR-138 and miR-let-7 in Cranial Neural Crest Cells. Stem Cells (2016) 34(11):2721–32. doi: 10.1002/stem.2421 27300003

[53] Boshnjaku V Shim KW Tsurubuchi T Ichi S Szany EV Xi G. Nuclear localization of folate receptor alpha: a new role as a transcription factor. Sci Rep (2012) 2:980. doi: 10.1038/srep00980 23243496 PMC3522071

[54] Hansen MF Greibe E Skovbjerg S Rohde S Kristensen AC Jensen TR. Folic acid mediates activation of the pro-oncogene STAT3 via the Folate Receptor alpha. Cell Signal (2015) 27(7):1356–68. doi: 10.1016/j.cellsig.2015.03.020 25841994

[55] Nawaz FZ Kipreos ET. Emerging roles for folate receptor FOLR1 in signaling and cancer. Trends Endocrinol Metabol: TEM (2022) 33(3):159–74. doi: 10.1016/j.tem.2021.12.003 PMC8923831 35094917

[56] Lin SY Lee WR Su YF Hsu SP Lin HC Ho PY. Folic acid inhibits endothelial cell proliferation through activating the cSrc/ERK 2/NF-kappaB/p53 pathway mediated by folic acid receptor. Angiogenesis (2012) 15(4):671–83. doi: 10.1007/s10456-012-9289-6 22843228

[57] Figini M Ferri R Mezzanzanica D Bagnoli M Luison E Miotti S. Reversion of transformed phenotype in ovarian cancer cells by intracellular expression of anti folate receptor antibodies. Gene Ther (2003) 10(12):1018–25. doi: 10.1038/sj.gt.3301962 12776159

[58] Varaganti P Buddolla V Lakshmi BA Kim YJ. Recent advances in using folate receptor 1 (FOLR1) for cancer diagnosis and treatment, with an emphasis on cancers that affect women. Life Sci (2023) 326:121802. doi: 10.1016/j.lfs.2023.121802 37244363

[59] Konner JA Bell-McGuinn KM Sabbatini P Hensley ML Tew WP Pandit-Taskar N. Farletuzumab, a humanized monoclonal antibody against folate receptor alpha, in epithelial ovarian cancer: a phase I study. Clin Cancer Res (2010) 16(21):5288–95. doi: 10.1158/1078-0432.CCR-10-0700 20855460

[60] Armstrong DK White AJ Weil SC Phillips M Coleman RL. Farletuzumab (a monoclonal antibody against folate receptor alpha) in relapsed platinum-sensitive ovarian cancer. Gynecol Oncol (2013) 129(3):452–8. doi: 10.1016/j.ygyno.2013.03.002 23474348

[61] Vergote I Armstrong D Scambia G Teneriello M Sehouli J Schweizer C. A randomized, double-blind, placebo-controlled, phase III study to assess efficacy and safety of weekly farletuzumab in combination with carboplatin and taxane in patients with ovarian cancer in first platinum-sensitive relapse. J Clin Oncol (2016) 34(19):2271–8. doi: 10.1200/JCO.2015.63.2596 27001568

[62] Spicer J Basu B Montes A Banerji U Kristeleit R Miller R. Safety and anti-tumour activity of the IgE antibody MOv18 in patients with advanced solid tumours expressing folate receptor-alpha: a phase I trial. Nat Commun (2023) 14(1):4180. doi: 10.1038/s41467-023-39679-9 37491373 PMC10368744

[63] Shimizu T Fujiwara Y Yonemori K Koyama T Sato J Tamura K. First-in-human phase 1 study of MORAb-202, an antibody-drug conjugate comprising farletuzumab linked to eribulin mesylate, in patients with folate receptor-α-positive advanced solid tumors. Clin Cancer Res (2021) 27(14):3905–15. doi: 10.1158/1078-0432.Ccr-20-4740 33926914

[64] Moore KN Borghaei H O'Malley DM Jeong W Seward SM Bauer TM. Phase 1 dose-escalation study of mirvetuximab soravtansine (IMGN853), a folate receptor alpha-targeting antibody-drug conjugate, in patients with solid tumors. Cancer (2017) 123(16):3080–7. doi: 10.1002/cncr.30736 PMC6896318 28440955

[65] O'Malley DM Matulonis UA Birrer MJ Castro CM Gilbert L Vergote I. Phase Ib study of mirvetuximab soravtansine, a folate receptor alpha (FRalpha)-targeting antibody-drug conjugate (ADC), in combination with bevacizumab in patients with platinum-resistant ovarian cancer. Gynecol Oncol (2020) 157(2):379–85. doi: 10.1016/j.ygyno.2020.01.037 32081463

[66] Matulonis UA Lorusso D Oaknin A Pignata S Dean A Denys H. Efficacy and safety of mirvetuximab soravtansine in patients with platinum-resistant ovarian cancer with high folate receptor alpha expression: results from the SORAYA study. J Clin Oncol (2023) 41(13):2436–45. doi: 10.1200/JCO.22.01900 PMC10150846 36716407

[67] Moore KN Oza AM Colombo N Oaknin A Scambia G Lorusso D. randomized trial of mirvetuximab soravtansine versus chemotherapy in patients with platinum-resistant ovarian cancer: primary analysis of FORWARD I. Ann Oncol (2021) 32(6):757–65. doi: 10.1016/j.annonc.2021.02.017 33667670

[68] Kershaw MH Westwood JA Parker LL Wang G Eshhar Z Mavroukakis SA. A phase I study on adoptive immunotherapy using gene-modified T cells for ovarian cancer. Clin Cancer Res (2006) 12(20 Pt 1):6106–15. doi: 10.1158/1078-0432.CCR-06-1183 PMC2154351 17062687

[69] Brown TA Byrd K Vreeland TJ Clifton GT Jackson DO Hale DF. Final analysis of a phase I/IIa trial of the folate-binding protein-derived E39 peptide vaccine to prevent recurrence in ovarian and endometrial cancer patients. Cancer Med (2019) 8(10):4678–87. doi: 10.1002/cam4.2378 PMC6712444 31274231

[70] Kalli KR Block MS Kasi PM Erskine CL Hobday TJ Dietz A. Folate receptor alpha peptide vaccine generates immunity in breast and ovarian cancer patients. Clin Cancer Res (2018) 24(13):3014–25. doi: 10.1158/1078-0432.CCR-17-2499 PMC6030477 29545464

[71] Banerjee S Michalarea V Ang JE Ingles Garces A Biondo A Funingana IG. A phase I trial of CT900, a novel alpha-folate receptor-mediated thymidylate synthase inhibitor, in patients with solid tumors with expansion cohorts in patients with high-grade serous ovarian cancer. Clin Cancer Res (2022) 28(21):4634–41. doi: 10.1158/1078-0432.CCR-22-1268 PMC9623233 35984704

[72] Lorusso PM Edelman MJ Bever SL Forman KM Pilat M Quinn MF. Phase I study of folate conjugate EC145 (Vintafolide) in patients with refractory solid tumors. J Clin Oncol (2012) 30(32):4011–6. doi: 10.1200/JCO.2011.41.4946 PMC4104289 23032618

[73] Naumann RW Coleman RL Burger RA Sausville EA Kutarska E Ghamande SA. PRECEDENT: a randomized phase II trial comparing vintafolide (EC145) and pegylated liposomal doxorubicin (PLD) in combination versus PLD alone in patients with platinum-resistant ovarian cancer. J Clin Oncol (2013) 31(35):4400–6. doi: 10.1200/JCO.2013.49.7685 24127448

[74] Sato S Itamochi H. Profile of farletuzumab and its potential in the treatment of solid tumors. OncoTargets Ther (2016) 9:1181–8. doi: 10.2147/OTT.S98242 PMC4789847 27022278

[75] Cheung A Opzoomer J Ilieva KM Gazinska P Hoffmann RM Mirza H. Anti-folate receptor alpha-directed antibody therapies restrict the growth of triple-negative breast cancer. Clin Cancer Res (2018) 24(20):5098–111. doi: 10.1158/1078-0432.CCR-18-0652 PMC6193548 30068707

[76] Spicer J Basu B Montes A Banerji U Kristeleit R Veal GJ. Abstract CT141: Phase 1 trial of MOv18, a first-in-class IgE antibody therapy for cancer. Cancer Res (2020) 80(16_Supplement). doi: 10.1158/1538-7445

[77] Ledermann JA Canevari S Thigpen T. Targeting the folate receptor: diagnostic and therapeutic approaches to personalize cancer treatments. Ann Oncol (2015) 26(10):2034–43. doi: 10.1093/annonc/mdv250 26063635

[78] Loebrich S Shen M Cohen E Payne G Chen Y Bogalhas M. Development and characterization of a neutralizing anti-idiotype antibody against mirvetuximab for analysis of clinical samples. AAPS J (2017) 19(4):1223–34. doi: 10.1208/s12248-017-0098-0 28534292

[79] Shah P Shlanksy-Goldberg R Martin L Nadolski G Hexner E Shamimi-Noori S. 431 First-in-human phase I clinical trial evaluating intraperitoneal administration of MOv19-BBz CAR T cells in patients with alpha folate receptor-expressing recurrent high grade serous ovarian cancer. J Immunother Cancer (2021) 9(Suppl 2):A461–A. doi: 10.1136/jitc-2021-SITC2021.431

[80] Ab O Whiteman KR Bartle LM Sun X Singh R Tavares D. IMGN853, a folate receptor-α (FRα)-targeting antibody-drug conjugate, exhibits potent targeted antitumor activity against FRα-expressing tumors. Mol Cancer Ther (2015) 14(7):1605–13. doi: 10.1158/1535-7163.Mct-14-1095 25904506

[81] Manzano A Ocana A. Antibody-drug conjugates: A promising novel therapy for the treatment of ovarian cancer. Cancers (2020) 12(8):2223. doi: 10.3390/cancers12082223 32784819 PMC7464539

[82] Cheng X Li J Tanaka K Majumder U Milinichik AZ Verdi AC. MORAb-202, an antibody-drug conjugate utilizing humanized anti-human FRalpha farletuzumab and the microtubule-targeting agent eribulin, has potent antitumor activity. Mol Cancer Ther (2018) 17(12):2665–75. doi: 10.1158/1535-7163.MCT-17-1215 30262588

[83] Furuuchi K Rybinski K Fulmer J Moriyama T Drozdowski B Soto A. Antibody-drug conjugate MORAb-202 exhibits long-lasting antitumor efficacy in TNBC PDx models. Cancer Sci (2021) 112(6):2467–80. doi: 10.1111/cas.14898 PMC8177789 33756060

[84] Cortes J O'Shaughnessy J Loesch D Blum JL Vahdat LT Petrakova K. Eribulin monotherapy versus treatment of physician's choice in patients with metastatic breast cancer (EMBRACE): a phase 3 open-label randomised study. Lancet (London England) (2011) 377(9769):914–23. doi: 10.1016/s0140-6736(11)60070-6 21376385

[85] Gerber HP Sapra P Loganzo F May C. Combining antibody-drug conjugates and immune-mediated cancer therapy: What to expect? Biochem Pharmacol (2016) 102:1–6. doi: 10.1016/j.bcp.2015.12.008 26686577

[86] Demetri GD Schöffski P Grignani G Blay JY Maki RG Van Tine BA. Activity of eribulin in patients with advanced liposarcoma demonstrated in a subgroup analysis from a randomized phase III study of eribulin versus dacarbazine. J Clin Oncol (2017) 35(30):3433–9. doi: 10.1200/JCO.2016.71.6605 28854066

[87] Sakai H Kawakami H Teramura T Onodera Y Somers E Furuuchi K. Folate receptor α increases chemotherapy resistance through stabilizing MDM2 in cooperation with PHB2 that is overcome by MORAb-202 in gastric cancer. Clin Trans Med (2021) 11(6):e454. doi: 10.1002/ctm2.454 PMC8167866 34185411

[88] Ab O Whiteman KR Bartle LM Sun X Singh R Tavares D. IMGN853, a folate receptor-alpha (FRalpha)-targeting antibody-drug conjugate, exhibits potent targeted antitumor activity against FRalpha-expressing tumors. Mol Cancer Ther (2015) 14(7):1605–13. doi: 10.1158/1535-7163.MCT-14-1095 25904506

[89] Oroudjev E Lopus M Wilson L Audette C Provenzano C Erickson H. Maytansinoid-antibody conjugates induce mitotic arrest by suppressing microtubule dynamic instability. Mol Cancer Ther (2010) 9(10):2700–13. doi: 10.1158/1535-7163.MCT-10-0645 PMC2976674 20937595

[90] Moore KN Martin LP O'Malley DM Matulonis UA Konner JA Vergote I. A review of mirvetuximab soravtansine in the treatment of platinum-resistant ovarian cancer. Future Oncol (London England) (2018) 14(2):123–36. doi: 10.2217/fon-2017-0379 29098867

[91] Martin LP Konner JA Moore KN Seward SM Matulonis UA Perez RP. Characterization of folate receptor alpha (FRalpha) expression in archival tumor and biopsy samples from relapsed epithelial ovarian cancer patients: A phase I expansion study of the FRalpha-targeting antibody-drug conjugate mirvetuximab soravtansine. Gynecol Oncol (2017) 147(2):402–7. doi: 10.1016/j.ygyno.2017.08.015 PMC6893864 28843653

[92] Stewart D Cristea M. Antibody-drug conjugates for ovarian cancer: current clinical development. Curr Opin Obstet Gynecol (2019) 31(1):18–23. doi: 10.1097/GCO.0000000000000515 30531606

[93] Moore KN Martin LP O'Malley DM Matulonis UA Konner JA Perez RP. Safety and activity of mirvetuximab soravtansine (IMGN853), a folate receptor alpha-targeting antibody-drug conjugate, in platinum-resistant ovarian, fallopian tube, or primary peritoneal cancer: A phase I expansion study. J Clin Oncol (2017) 35(10):1112–8. doi: 10.1200/JCO.2016.69.9538 PMC5559878 28029313

[94] Moore KN Vergote I Oaknin A Colombo N Banerjee S Oza A. FORWARD I: a Phase III study of mirvetuximab soravtansine versus chemotherapy in platinum-resistant ovarian cancer. Future Oncol (London England) (2018) 14(17):1669–78. doi: 10.2217/fon-2017-0646 29424243

[95] Ponte JF Ab O Lanieri L Lee J Coccia J Bartle LM. Mirvetuximab soravtansine (IMGN853), a folate receptor alpha-targeting antibody-drug conjugate, potentiates the activity of standard of care therapeutics in ovarian cancer models. Neoplasia (2016) 18(12):775–84. doi: 10.1016/j.neo.2016.11.002 PMC5126132 27889646

[96] Gilbert L Oaknin A Matulonis UA Mantia-Smaldone GM Lim PC Castro CM. Safety and efficacy of mirvetuximab soravtansine, a folate receptor alpha (FRalpha)-targeting antibody-drug conjugate (ADC), in combination with bevacizumab in patients with platinum-resistant ovarian cancer. Gynecol Oncol (2023) 170:241–7. doi: 10.1016/j.ygyno.2023.01.020 36736157

[97] Tarantino P Ricciuti B Pradhan SM Tolaney SM. Optimizing the safety of antibody-drug conjugates for patients with solid tumours. Nat Rev Clin Oncol (2023) 20(8):558–76. doi: 10.1038/s41571-023-00783-w 37296177

[98] Hendershot A Slabaugh M Riaz KM Moore KN O'Malley DM Matulonis U. Strategies for prevention and management of ocular events occurring with mirvetuximab soravtansine. Gynecol Oncol Rep (2023) 47:101155. doi: 10.1016/j.gore.2023.101155 37102083 PMC10123335

[99] Parslow AC Parakh S Lee FT Gan HK Scott AM. Antibody-drug conjugates for cancer therapy. Biomedicines (2016) 4(3):4764. doi: 10.3390/biomedicines4030014 PMC5344263 28536381

[100] Eaton JS Miller PE Mannis MJ Murphy CJ. Ocular adverse events associated with antibody-drug conjugates in human clinical trials. J Ocular Pharmacol Ther (2015) 31(10):589–604. doi: 10.1089/jop.2015.0064 PMC4677113 26539624

[101] Matulonis UA Birrer MJ O'Malley DM Moore KN Konner J Gilbert L. Evaluation of prophylactic corticosteroid eye drop use in the management of corneal abnorMalities induced by the antibody-drug conjugate mirvetuximab soravtansine. Clin Cancer Res (2019) 25(6):1727–36. doi: 10.1158/1078-0432.CCR-18-2474 30413525

[102] Corbelli E Miserocchi E Marchese A Giuffrè C Berchicci L Sacconi R. Ocular toxicity of mirvetuximab. Cornea (2019) 38(2):229–32. doi: 10.1097/ICO.0000000000001805 30379722

[103] Song DG Ye Q Poussin M Chacon JA Figini M Powell DJ Jr. Effective adoptive immunotherapy of triple-negative breast cancer by folate receptor-alpha redirected CAR T cells is influenced by surface antigen expression level. J Hematol Oncol (2016) 9(1):56. doi: 10.1186/s13045-016-0285-y 27439908 PMC4955216

[104] Song DG Ye Q Poussin M Harms GM Figini M Powell DJ Jr. CD27 costimulation augments the survival and antitumor activity of redirected human T cells in vivo. Blood (2012) 119(3):696–706. doi: 10.1182/blood-2011-03-344275 22117050

[105] Huang J Kerstann KW Ahmadzadeh M Li YF El-Gamil M Rosenberg SA. Modulation by IL-2 of CD70 and CD27 expression on CD8+ T cells: importance for the therapeutic effectiveness of cell transfer immunotherapy. J Immunol (2006) 176(12):7726–35. doi: 10.4049/jimmunol.176.12.7726 PMC1532931 16751420

[106] Song DG Ye Q Carpenito C Poussin M Wang LP Ji C. *In vivo* persistence, tumor localization, and antitumor activity of CAR-engineered T cells is enhanced by costimulatory signaling through CD137 (4-1BB). Cancer Res (2011) 71(13):4617–27. doi: 10.1158/0008-5472.CAN-11-0422 PMC4140173 21546571

[107] Liang Z Dong J Yang N Li SD Yang ZY Huang R. Tandem CAR-T cells targeting FOLR1 and MSLN enhance the antitumor effects in ovarian cancer. Int J Biol Sci (2021) 17(15):4365–76. doi: 10.7150/ijbs.63181 PMC8579462 34803504

[108] Farran B Pavitra E Kasa P Peela S Rama Raju GS Nagaraju GP. Folate-targeted immunotherapies: Passive and active strategies for cancer. Cytokine Growth Factor Rev (2019) 45:45–52. doi: 10.1016/j.cytogfr.2019.02.001 30770191

[109] Malonis RJ Lai JR Vergnolle O. Peptide-based vaccines: current progress and future challenges. Chem Rev (2020) 120(6):3210–29. doi: 10.1021/acs.chemrev.9b00472 PMC7094793 31804810

[110] Kim DK Lee TV Castilleja A Anderson BW Peoples GE Kudelka AP. Folate binding protein peptide 191-199 presented on dendritic cells can stimulate CTL from ovarian and breast cancer patients. Anticancer Res (1999) 19(4b):2907–16. 10652572

[111] Tochowicz A Dalziel S Eidam O O'Connell JD 3rd Griner S Finer-Moore JS. Development and binding mode assessment of N-[4-[2-propyn-1-yl[(6S)-4,6,7,8-tetrahydro-2-(hydroxymethyl)-4-oxo-3H-cyclopenta[g]quinazolin-6-yl]amino]benzoyl]-l-gamma-glutamyl-D-glutamic acid (BGC 945), a novel thymidylate synthase inhibitor that targets tumor cells. J Med Chem (2013) 56(13):5446–55. doi: 10.1021/jm400490e PMC3880649 23710599

[112] Reddy JA Nelson M Dircksen C Vetzel M Johnson T Cross V. Pre-clinical studies of EC2629, a highly potent folate- receptor-targeted DNA crosslinking agent. Sci Rep (2020) 10(1):12772. doi: 10.1038/s41598-020-69682-9 32728172 PMC7391724

[113] Peethambaram PP Hartmann LC Jonker DJ de Jonge M Plummer ER Martin L. A phase I pharmacokinetic and safety analysis of epothilone folate (BMS-753493), a folate receptor targeted chemotherapeutic agent in humans with advanced solid tumors. Invest New Drugs (2015) 33(2):321–31. doi: 10.1007/s10637-014-0171-9 25380635

[114] Vergote I Leamon CP. Vintafolide: a novel targeted therapy for the treatment of folate receptor expressing tumors. Ther Adv Med Oncol (2015) 7(4):206–18. doi: 10.1177/1758834015584763 PMC4480526 26136852

[115] Moore KN O'Malley DM Vergote I Martin LP Gonzalez-Martin A Malek K. Safety and activity findings from a phase 1b escalation study of mirvetuximab soravtansine, a folate receptor alpha (FRα)-targeting antibody-drug conjugate (ADC), in combination with carboplatin in patients with platinum-sensitive ovarian cancer. Gynecol Oncol (2018) 151(1):46–52. doi: 10.1016/j.ygyno.2018.07.017 30093227

[116] O'Malley DM Matulonis UA Birrer MJ Castro CM Gilbert L Vergote I. Phase Ib study of mirvetuximab soravtansine, a folate receptor alpha (FRα)-targeting antibody-drug conjugate (ADC), in combination with bevacizumab in patients with platinum-resistant ovarian cancer. Gynecol Oncol (2020) 157(2):379–85. doi: 10.1016/j.ygyno.2020.01.037 32081463

