# Corrigendum: Therapeutic strategies targeting folate receptor α for ovarian cancer

**DOI:** 10.3389/fimmu.2024.1403324

**Published:** 2024-04-17

**Authors:** Jia Mai, Limei Wu, Ling Yang, Ting Sun, Xiaojuan Liu, Rutie Yin, Yongmei Jiang, Jinke Li, Qintong Li

**Affiliations:** ^1^ Department of Laboratory Medicine, Obstetrics & Gynecology and Pediatrics, West China Second University Hospital, Key Laboratory of Birth Defects and Related Diseases of Women and Children, Ministry of Education, Development and Related Diseases of Women and Children Key Laboratory of Sichuan Province, Center of Growth, Metabolism and Aging, State Key Laboratory of Biotherapy and Collaborative Innovation Center of Biotherapy, Sichuan University, Chengdu, Sichuan, China; ^2^ Department of Obstetrics and Gynecology, Chengdu Second People’s Hospital, Chengdu, Sichuan, China; ^3^ Department of Clinical Laboratory, The first Affiliated Hospital of Zhengzhou University, Zhengzhou, China

**Keywords:** ovarian cancer, folate receptor α, FOLR1, mirvetuximab soravtansine, MIRV, Elahere, antibody-drug conjugate, ADC

In the published article, there was an error in **Figure 1** as published. PCTF is a symporter that transport H+ ion together with the folate transporter, we made a mistake with the direction of one arrow, by making it like PCFT is portrayed as an antiporter. The corrected **Figure 1** and its caption appear below.

**Figure 1:**
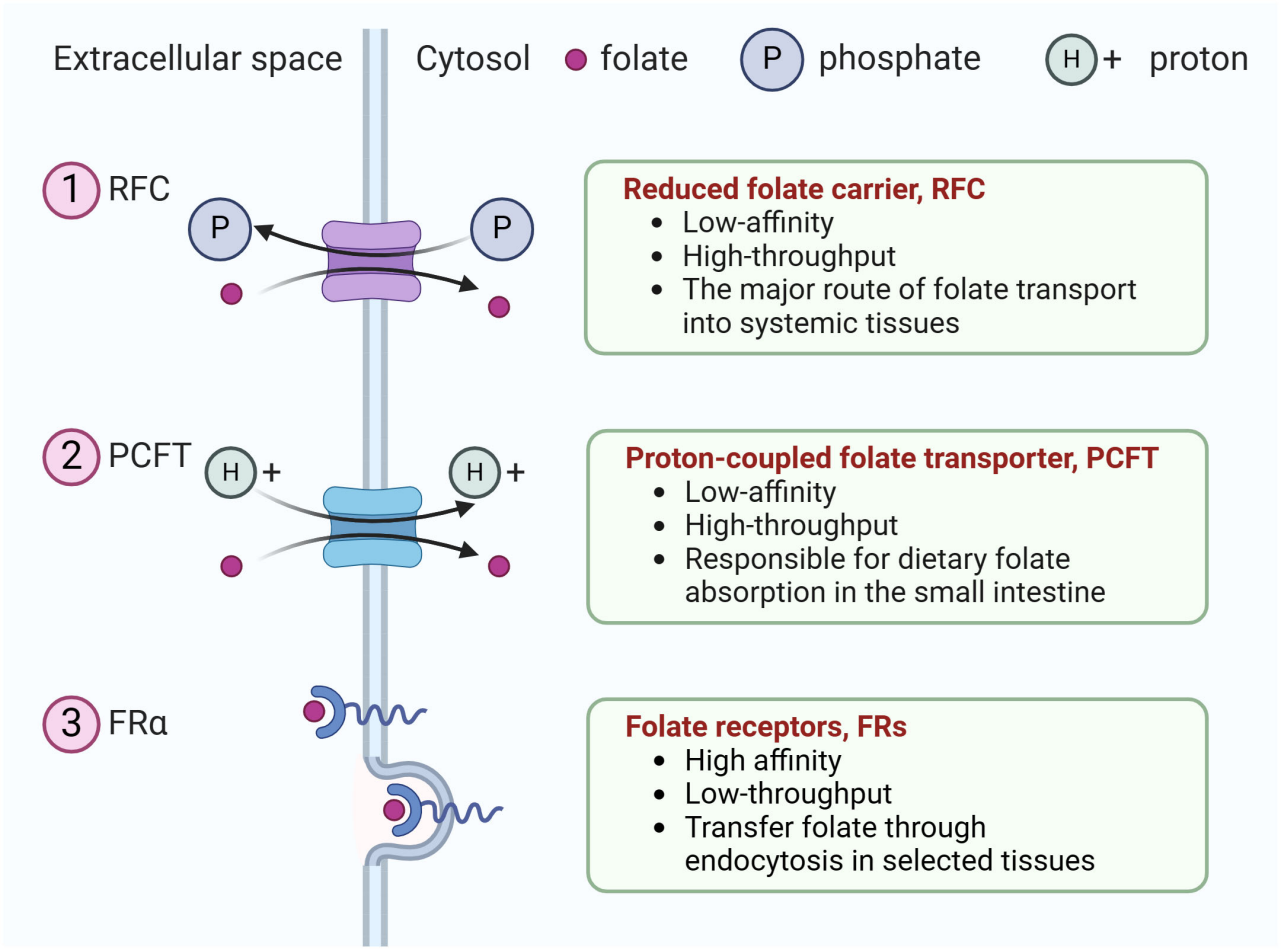
The three types of folate transporters. The uptake of extracellular folate is achieved mainly through three types of folate transporters. (1) RFC, an anion antiporter that uses a gradient of higher organic phosphate in the cell to transport folate into the cell while transporting organic phosphate out of the cell, (2) PCFT, a proton-coupled transporter, (3) folate receptor family (only FRα is shown). They transfer folate through endocytosis in selected tissues.

The authors apologize for this error and state that this does not change the scientific conclusions of the article in any way. The original article has been updated.

